# Biofouling of a unionid mussel by dreissenid mussels in nearshore zones of the Great Lakes

**DOI:** 10.1002/ece3.9557

**Published:** 2022-12-13

**Authors:** James H. Larson, Sean W. Bailey, Mary Anne Evans

**Affiliations:** ^1^ U.S. Geological Survey, Upper Midwest Environmental Sciences Center La Crosse Wisconsin USA; ^2^ U.S. Geological Survey, Great Lakes Science Center Ann Arbor Michigan USA

**Keywords:** biofouling, dreissenid mussels, Great Lakes, Unionid mussels, zebra mussel

## Abstract

In North America, native unionid mussels are imperiled due to factors such as habitat degradation, pollution, and invasive species. One of the most substantial threats is that posed by dreissenid mussels, which are invasive mussels that attach to hard substrates including unionid shells and can restrict movement and feeding of unionids. This dreissenid mussel biofouling of unionids varies spatially in large ecosystems, such as the Great Lakes, with some areas having low enough biofouling to form effective refugia where unionid mussels might persist. Here, we measured biofouling on mussels suspended in cages over the growing season (generally first week in June to last week of August) over 3 years in nearshore areas in Lake Erie (2014–2016), Lake Michigan (Grand Traverse Bay, 2015 and Green Bay, 2016), and Lake Huron (2015). Biofouling varied substantially by years within Lake Erie, with increasingly higher biofouling rates each year. Although dreissenid mussels are present throughout these lakes, we observed very low biofouling in Grand Traverse Bay (Lake Michigan) and Saginaw Bay (Lake Huron), with no dreissenid mussels in 8 of 9 sites across these two bays. Sampling in the rivermouth of the Fox River (Wisconsin) and the Maumee River (Ohio) both showed very high biofouling in areas adjacent to the outlet of these tributaries into Green Bay and Maumee Bay (Lake Erie), respectively. These watersheds are dominated by agriculture, and we would expect high growth of primary producers (i.e., mussel food) and primary consumers (unionids and zebra mussels) in these areas compared to the other sampled bays or the open waters of the Great Lakes.

## Graphical abstract

Biofouling rates in open water, western Lake Erie sites (LE) were much higher than biofouling rates in open water sites of Saginaw Bay or Grand Traverse Bay. The highest biofouling rates in Maumee Bay (Lake Erie) and Green Bay (Lake Michigan) occurred within 10 km of where the Maumee and Fox Rivers entered these lakes. A moderate positive correlation between biofouling and mussel growth suggests both dreissenid and unionid mussels do better in the same habitats.

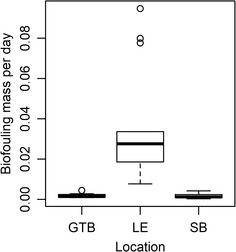

## 1 INTRODUCTION

Freshwater bivalves of North America (family Unionidae) comprise one of the most imperiled taxa in the world, with perhaps 30–35 species extinct and many of the remaining species considered vulnerable or endangered (Haag & Williams, 2013; Ricciardi et al., 1998). Among the threats to unionids, few are more concerning than invasive mussels in the *Dreissena* genus (Zebra mussels, *D. polymorpha* and quagga mussels, *D. rostriformis bugensis*), hereafter referred to as dreissenid mussels (Ricciardi et al., 1998). The primary cause of dreissenid mussel effects on unionid mussels is their heavy biofouling on unionid mussel shells, which can interfere with feeding and movement (Conn & Conn, 1993; Schloesser et al., 1996). The biofouling by dreissenid mussels may also increase biofouling by other organisms on unionid mussel shells (Conn & Conn, 1993; Nakano & Strayer, 2014; Ozersky et al., 2011). Lethal biofouling by dreissenid mussels on unionid mussels has usually been estimated in terms of population density (i.e., # per unionid; Ricciardi et al., 1996). Dreissenid mussel infestation greater than 100 individuals per unionid mussel (or *a* > 1.0 dreissenid mussel to unionid mussel mass ratio) appears to result in lethal conditions (Burlakova et al., 2014; Ricciardi et al., 1995, 1996), but even densities lower than this are harmful (Baker & Hornbach, 1997).

In large ecosystems with substantial environmental gradients, such as the Laurentian Great Lakes, there are often spatial and temporal gradients in the intensity of biofouling by dreissenid mussels (Burlakova et al., 2014; Fraleigh et al., 1992; Larson, Richardson, Kennedy, et al., 2016; Sherman et al., 2013). These gradients in biofouling could result in differential effects to unionid mussels in terms of their ability to grow or survive (Burlakova et al., 2014; Crail et al., 2011; McGoldrick et al., 2009). Many studies of dreissenid mussel biofouling on unionid mussels have measured in situ biofouling on wild individuals, which is important for understanding ecosystem effects (Burlakova et al., 2014). However, biofouling densities on wild mussels reflect many interactions between the population of biofouling organisms, the biology of the unionid mussel, and the abiotic environment (e.g., sediment characteristics) and are limited to areas where unionid mussels persist. For example, Burlakova et al. (2014) sampled 26 different species of mussels and concluded among‐species differences in biofouling susceptibility were low, but obviously only surviving species‐habitat combinations could be sampled. Burlakova et al. (2014) and other studies that measure biofouling on wild individuals do not estimate biofouling intensity in locations where wild individuals are absent, creating a biased understanding of the spatial distribution of biofouling. In addition, zebra mussel densities in Lake Erie have declined from their peak early in the invasion cycle (Karatayev et al., 2014) and have been replaced by quagga mussels, which do not colonize unionid mussels with the same intensity as zebra mussels (Conn & Conn, 1993). This indicates that there may be areas where biofouling rates have declined and unionid mussels could successfully recolonize. Mussel conservation in many parts of North America has included artificial propagation as a method to facilitate recolonization of native unionid mussels more rapidly than natural expansion would occur, but it would be ideal to establish that biofouling rates are indeed low in areas where artificial propagation might occur (Patterson et al., 2018).

A complementary approach to the observations of naturally occurring conditions is to create semi‐artificial, but standardized conditions, which can be extended systematically across large temporal and spatial gradients. For example, colonization rates of aquatic invertebrates are often measured using standardized surfaces (Hester & Dendy, 1962). Standardized, semi‐artificial approaches such as this often create less realistic conditions, so they complement rather than replace in situ observations. Still, standardized methods can be designed and implemented in ways that measure environmental gradients in approaches that would be impossible with solely observational studies.

In the case of unionid mussel biofouling, Larson, Richardson, Kennedy, et al. (2016) used caged unionid mussels to measure spatial variation in biofouling in western Lake Erie during the 2014 growing season. These measurements covered an enormous range of conditions in western Lake Erie and found biofouling rates (primarily by dreissenid mussels) varied by 3 orders of magnitudes, with some sites having very little or no dreissenid mussel biofouling (although dreissenid mussels accounted for most of the variation in biofouling across all sites). That study identified large areas where unionid mussels might persist (due to both low biofouling and ideal substrate), that were outside the shallow, nearshore areas usually sampled for remnant unionid mussel populations (e.g., see Figure 1 of Burlakova et al., 2014). Although Larson, Richardson, Kennedy, et al. (2016) highlighted the potential value of a more standardized approach applied across environmental gradients that otherwise could not be sampled, the spatial extent was limited to the western basin of Lake Erie and to a single growing season.

**FIGURE 1:**
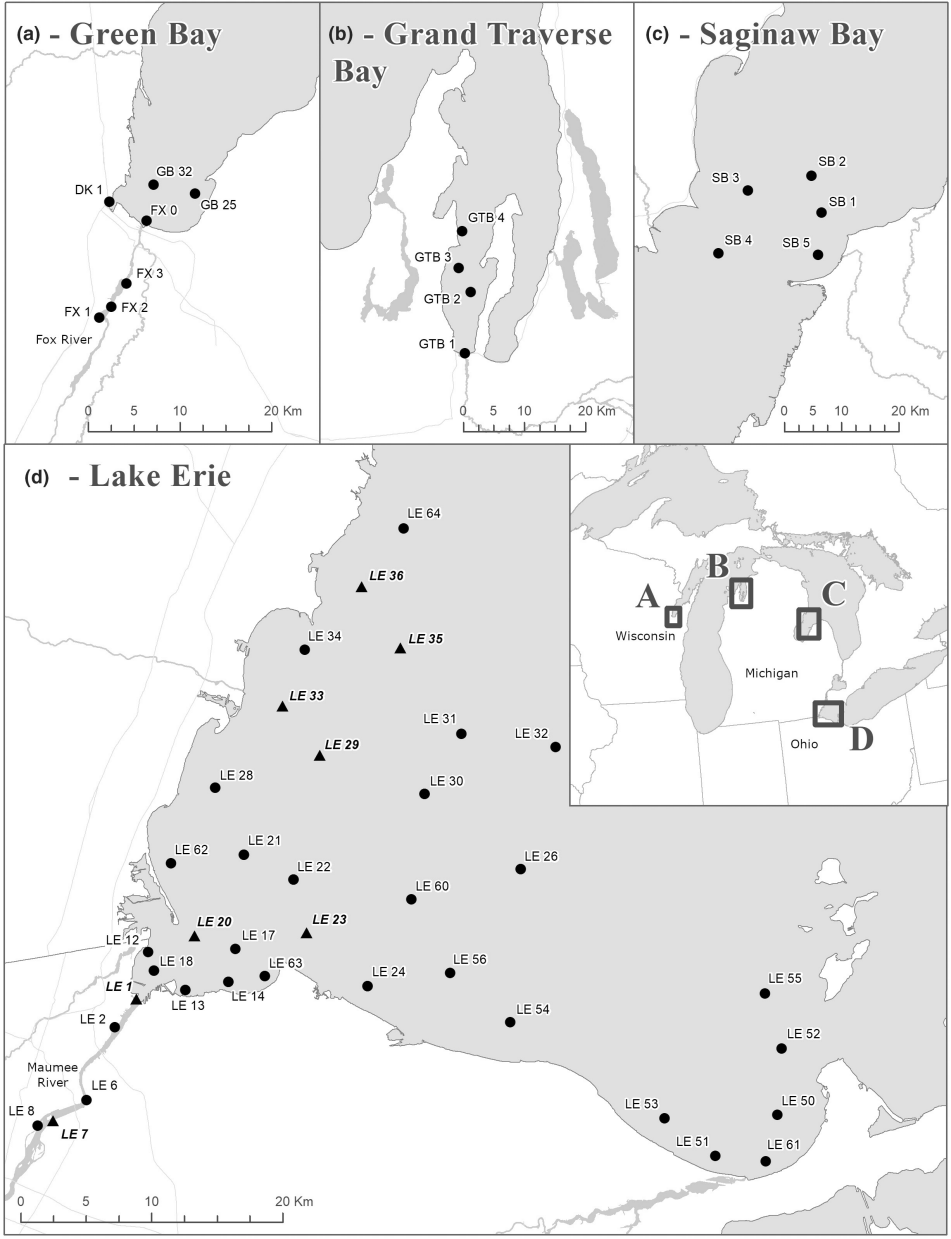
Map of locations where unionid biofouling was measured. Sites were sampled for 1 year in (a) Green Bay (2016), (b) Grand Traverse Bay (2015), and (c) Saginaw Bay (2015). A total of 3 years of sampling occurred in (d) western Lake Erie sites (2014–2016), but most sites were only sampled 1 or 2 times during this period. Sites marked with a triangle were sampled every year.

Here, we report on additional years of biofouling data using the same methods in western Lake Erie and other Great Lakes embayments. Our objectives were to assess (1) the degree to which overall biofouling rates vary annually in Lake Erie and (2) whether biofouling rates are similar across diverse Great Lakes nearshore areas. As a third objective, we then assessed whether biofouling varied in response to two important environmental characteristics that varied strongly among our sites (temperature and depth) and that are known to influence dreissenid mussel growth and recruitment (Karatayev et al., 2013, 2018).

## 2 METHODS

### 2.1 Study sites

Study sites were located in four Great Lakes nearshore areas (Figure 1). Sites in Lake Erie were previously described by Larson, Richardson, Kennedy, et al. (2016), Larson et al. (2018). Briefly, these sites were selected to cover a gradient in habitat conditions starting at the mouth of the Maumee River (hereafter, Maumee rivermouth), moving into Maumee Bay and then spreading across all of the western basin (Larson, Richardson, Evans, et al., 2016). Biofouling results for 37 sites sampled in 2014 were previously reported by Larson, Richardson, Kennedy, et al. (2016); in 2015 and 2016, a subset of the original 2014 sites was sampled and these data are first reported in the current manuscript.

Sites in Grand Traverse Bay (Lake Michigan, Michigan; 4 sites) and Saginaw Bay (Lake Huron, Michigan; 5 sites) were sampled in 2015. Situated in locations where other U.S. Geological Survey (USGS) water‐quality monitoring was occurring, these sites were selected to capture a gradient in water mass mixing (between river/bay water sources and the open waters of Lake Michigan and Lake Huron, respectively). Sites in Green Bay (Lake Michigan, Wisconsin; 7 sites) were sampled in 2016 and were situated to match ongoing USGS water quality experiments in the Fox River rivermouth and Duck Creek rivermouth (tributaries to Green Bay; Larson et al., 2019, 2020) and sites in Green Bay itself that were sampled for water‐quality monitoring by a local multi‐agency effort (known locally as NewWater).

### 2.2 Mussel cages

At each site, we deployed mussel cages with hatchery‐raised *Lampsilis siliquoidea*. *L. siliquoidea* are a native, formerly common species in the Great Lakes and the surrounding basin and still persist in Lake Erie and Lake St. Claire (Crail et al., 2011; Lucy et al., 2013). Deployments began in early summer (usually the 1st week of June) and retrieval took place in late summer (usually the last week of August). Dreissenid mussel veligers appear to occur in repeated waves within Lake Erie, but our sampling window probably covered the period when most veliger release occurs (Fraleigh et al., 1992).

Mussel cages consisted of plastic‐coated mesh with diamond‐shaped openings (maximum width = 1.4 cm). The mesh wrapped around two PVC drain caps, forming a cylindrical shape ~21 cm in height and 10.8 cm in diameter. Mesh was held in place with stainless steel hose clamps to form the mussel cage. At each site, a single cage was affixed to a rope anchored to a cement block (~13 kg) and suspended by a submerged buoy. This arrangement allowed suspension of the cages approximately halfway between the buoy and the cement block, about 1 m off the substrate. At most sites, the first cement block was tied to a second cement block to form a dual anchoring system that facilitated surface retrieval. In a few cases, cages could not be recovered by grappling from the surface and divers assisted in retrievals. A few of the cage anchors in the Maumee rivermouth were tied directly to bars welded into place along the seawall (sites LE1‐LE8).

### 2.3 Unionid mussels

Mussels used in this study varied in source by year. During 2014 and 2015, mussels were provided by Missouri State University's Mussel Culture Lab (Prof. Chris Barnhart). Gravid females for these cohorts came from the Silver Fork of Perch Creek (Missouri) with *Micropterus salmoides* as the glochidial host fish. These mussels had a median initial length of 28.5 mm and 46.7 mm in 2014 and 2015, respectively (Larson et al., 2022). In May 2015, gravid females were collected from a small lake in southern Michigan (42.3142678°N, 84.079889°W). These gravid females were propagated with *M. salmoides* as the glochidial host fish at the Columbus (Ohio) Zoo's freshwater mussel research facility (G. Thomas Watters, curator). Offspring from these gravid females were used in 2016 mussel cages. The 2016 mussels had a median initial length of 30.2 mm.

Prior to placing mussels into cages, mussels were depurated for 2 weeks in synthetic freshwater (i.e., purified water to which naturally occurring ions have been added) and visually inspected for any form of illness (by the individuals who propagated the mussels). Individuals were marked with an electric grinding tool and two (2015) or three (2014, 2016) individuals were placed in each cage. Only two individuals were placed in cages in 2015 because the mussels were larger and we hoped to avoid crowding that occurred in a previous version of this study in 2013 (Larson, Richardson, Evans, et al., 2016). At the end of the study, no unionid mussels were gravid, and most were too small to show sexual dimorphism in body shape (based on data provided by Prof. Chris Barnhart, written comm., 2016). Growth of these mussels was estimated by measuring the dimensions (length, width, height) before and after deployment, using the protocol described in Larson et al. (2014).

### 2.4 Measuring biofouling rate

We estimated biofouling by measuring wet weight of unionid mussels before and after removal of biofouling organisms. Dry weights could not be measured because animals were also being sampled for fatty acids, glycogen, and stable isotopes (as in Larson, Richardson, Evans, et al., 2016). Most biofouling organisms were dreissenid mussels, although we did not (and could not) distinguish between *Dreissenia bugensis* and *Dreissenia polymorpha* (Grigorovich et al., 2008). Hand scrubbing achieved removal of biofouling in most cases, but some traces of byssal threads could not be completely removed. We took wet weight after repeated blotting (both before and after removal of biofouling) to the nearest 0.01 g on a balance (OHAUS CT‐200‐5) with daily calibration checks. While blotting, we attempted to remove water retained in the mantle cavity. The mass of biofouling at the end of the exposure period was divided by the total number of days unionid mussels were deployed to normalize for differences in exposure time among different sampling years and locations. Exposures ranged from 81–105 days, with periods >90 days usually occurring because of weather‐related delays or the need to arrange for diver assistance in retrieving cages.

### 2.5 Water temperature

Water temperature data loggers (iButton Thermochron loggers; Maxim Integrated) were deployed with the caged unionid mussels in waterproof plastic containers (contact cases sealed with silicon), as described in Larson et al. (2018). To recap this methodology, these sensors record temperatures every 2 h while deployed. Instead of simply using average temperatures, we calculated degree days (Chezik et al., 2014). Degree days are useful for calculating the amount of ambient thermal energy that an animal has experienced, which is directly related to growth and other metabolic processes in ectotherms (Trudgill et al., 2005). Degree days have been used in a wide variety of applications to relate temperature to growth (Chezik et al., 2014; Trudgill et al., 2005). For each day, the following equation is used to calculate the degree days (DD) for that day:

$$\mathrm{DD}=\left[\frac{T\max-T\min}{2}\right]-T_{x}$$

where *T*_*x*_ is the threshold temperature under consideration (e.g., 0°C or 5°C) and *T*_max_ and *T*_min_ are the daily maximum and minimum temperatures, respectively. Negative daily DD estimates are discarded, and the positive daily DD estimates are summed for the deployment period. We calculated DDs for a *T*_*x*_ of 0°C, 5°C, 10°C, 12°C, 15°C, 20°C, and 25°C. For statistical analysis, we used DD15 to represent the effects of water temperature, both because it was highly correlated to other choices of temperature threshold and because this is close to the threshold temperature for growth in dreissenid mussels (Neumann et al., 1993). We found strong correlations between DD15 and other thresholds for estimating DDs (Pearson's *r* > .7).

### 2.6 Statistical analysis

We calculated descriptive site statistics by estimating the median, mean, and standard deviation of all mussels in each cage using R (R Development Core Team, 2019). Correlation between unionid mussel growth rate (volume increase per day) and biofouling rate (g per day) was estimated using a correlation coefficient at the individual level. We used Spearman's rho because it does not make distributional assumptions.

Differences in biofouling between different groups of unionid mussels were assessed using the mean difference approach described in McCarthy (2007), which uses a Bayesian methodology to estimate the differences in distribution among categories. This was implemented using JAGS and the R package rjags (Plummer, 2003). Detailed examples of the code and data are available online. Essentially, in this approach, the distribution of each category is estimated using an iterative approach, and for each iteration, the difference between means is calculated. The magnitude and variability of the difference can then be estimated from all these iterations. Differences between years (at sites sampled repeated in Lake Erie), bays, and habitat types (rivermouth vs. bay) were examined using this method. Biofouling data were not normally distributed (as evidenced by large differences between median and mean and visual inspection of plots of the data), so all biofouling rate data were log‐transformed prior to comparing distributions. Biofouling rates in Grand Traverse Bay and Saginaw Bay were only compared to “open‐water” sites in Lake Erie during the same year, as none of the Grand Traverse Bay or Saginaw Bay sites occurred near a large rivermouth, and were in deeper water (>5 m) than sites sampled from Green Bay or Maumee Bay (<4 m). Sites in the river‐to‐lake transition were sampled in both Green Bay and Maumee Bay, so these were compared visually, oriented by their distance from the outlet of the Fox River, Duck Creek, or Maumee River rivermouths.

We also estimated correlation coefficients between degree days, depth, and the biofouling rate to assess how much these environmental characteristics were related to biofouling rate.

## 3 RESULTS

### 3.1 Different measurements of biofouling co‐vary

**FIGURE 2:**
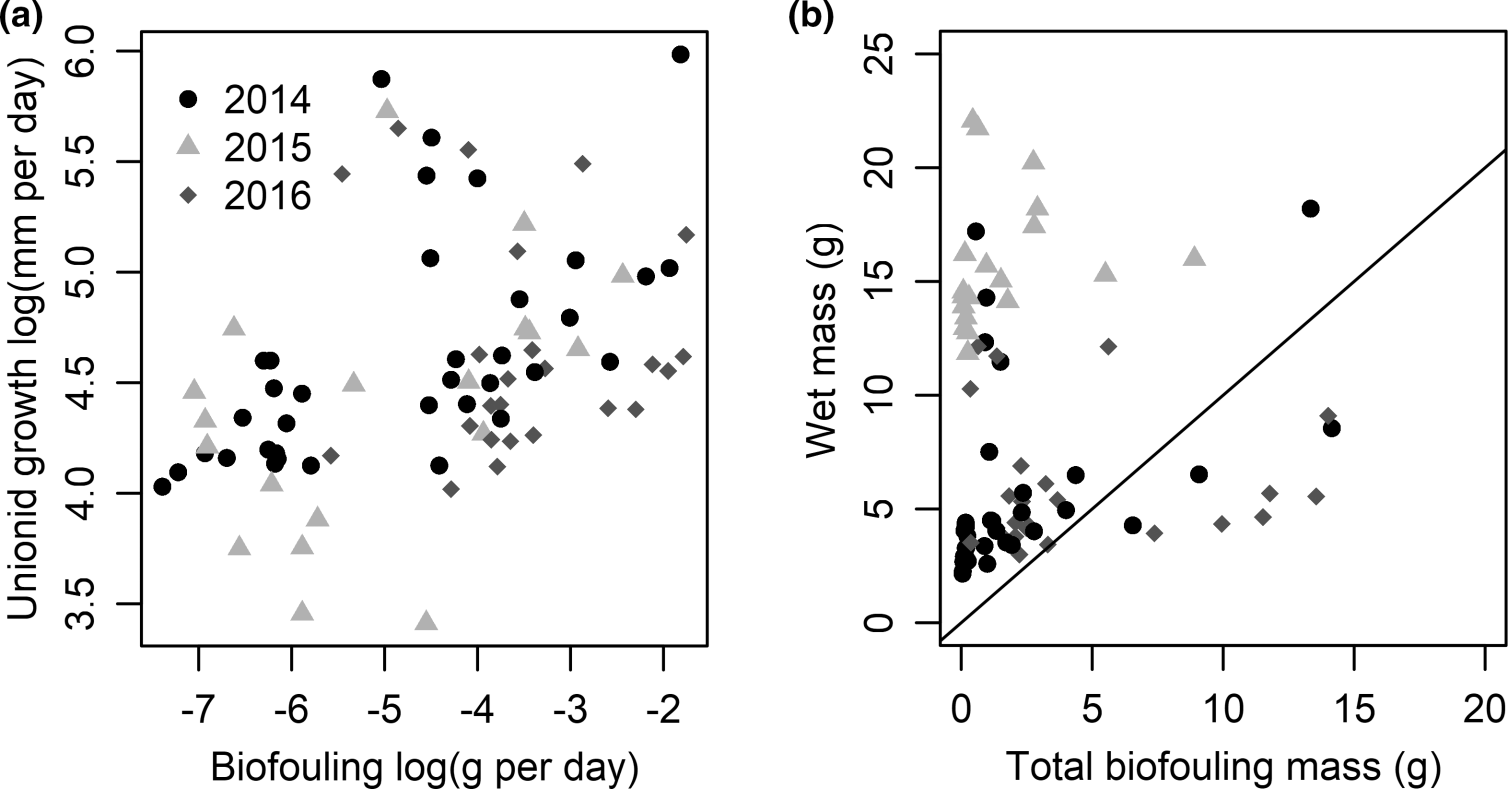
(a) Association between growth rate of unionid mussels (in units of volume increase per day) and biofouling rate (grams of accumulation per day). (b) Association between final wet mass of unionid mussels and final wet mass of biofouling (both in grams). Mussels were held in cages at sites in Lake Michigan, Lake Huron, and Lake Erie from 2014–2016. Two or three individual mussels were held in each cage. Each point is the median value for the cage. Panel (b) includes a 1:1 line showing that most unionid mussels were heavier than the mass of biofouling organisms.

The mean ratio of biofouling mass to unionid mass was >1 in nine cages over the course of the three‐year study, all from cages within Green Bay or Maumee Bay near the mouth of the Fox River and the Maumee River, respectively (Table S1). Estimating biofouling as a ratio or percentage of the unionid mussel body mass was strongly correlated with estimating the biofouling rate as simply the daily increase in mass (Figure S1, Pearson's *r* = .92). This indicates that the variation in surface area between our differently sized mussels had little influence on the amount of total biofouling that could occur on the individual unionid mussels. Therefore, we have focused hereafter on biofouling in units of mass increase per day. There was a moderate positive correlation between median cage biofouling rate (g day^−1^) and median cage growth rate of the mussels (daily increase in volume; Spearman's *ρ* = 0.48; Figure 2a). This correlation differed somewhat by year; Spearman's *ρ* was 0.62 for 2014, 0.48 for 2015, and 0.13 for 2016. A weak correlation was found between the final mussel wet mass and the final biofouling wet mass (Spearman's *ρ* 0.20; Figure 2b). The relationship between final mass and final biofouling wet mass was weakened partially because of very large differences in the initial sizes of the unionid mussels. Within years Spearman's *ρ* is 0.64 for 2014, 0.51 for 2015, and 0.06 for 2016.

### 3.2 Assessing the degree of temporal variation in biofouling in Lake Erie

**FIGURE 3:**
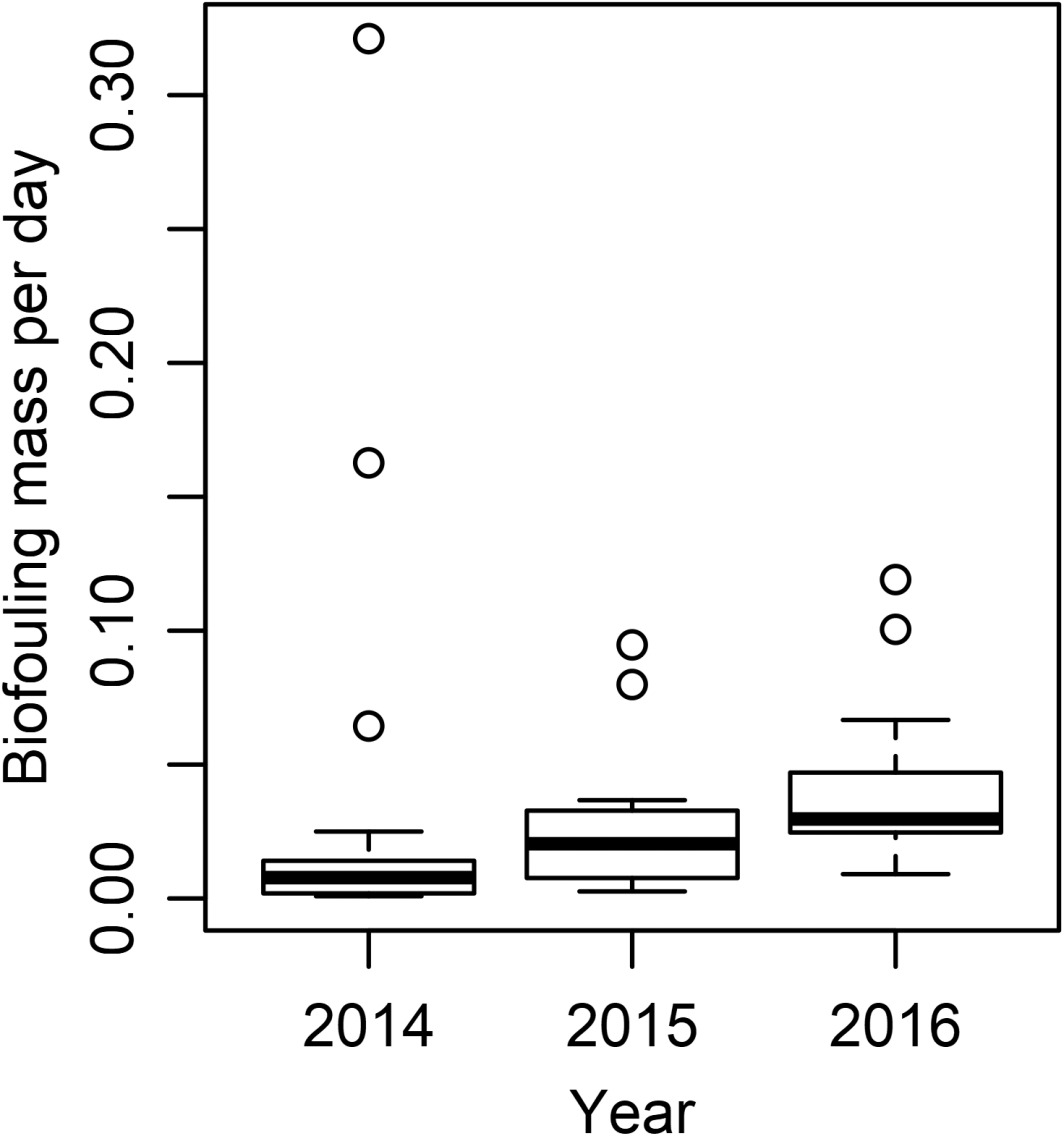
Box‐and‐whisker plot showing biofouling rates (g day^−1^) of individual unionid mussels deployed at sites sampled over 3 years (LE1, LE20, LE23, LE29, LE33, LE35, LE36, and LE7) during 2014, 2015, and 2016 field seasons. The 95% credible interval of the difference between years had a magnitude >0 with higher biofouling in each year.

A total of eight sites were sampled across all 3 years in Lake Erie (sites LE1, LE7, LE20, LE23, LE29, LE33, LE35, and LE36). Grouping these sites together, median biofouling rate was 0.0078 g day^−1^ in 2014, 0.0204 g day^−1^ in 2015, and 0.0297 g day^−1^ in 2016 (Figure 3), differences that appear to be substantial enough to indicate increases from year to year over the course of the study (Table S2). Degree days above 15°C (DD15) also changed at these sites with median DD15 being 651.5 in 2014, 689.5 in 2015, and 831.8 in 2016, and the role of temperature is explored in more detail below.

### 3.3 Assessing variation in biofouling between Lake Erie and other nearshore areas

**FIGURE 4:**
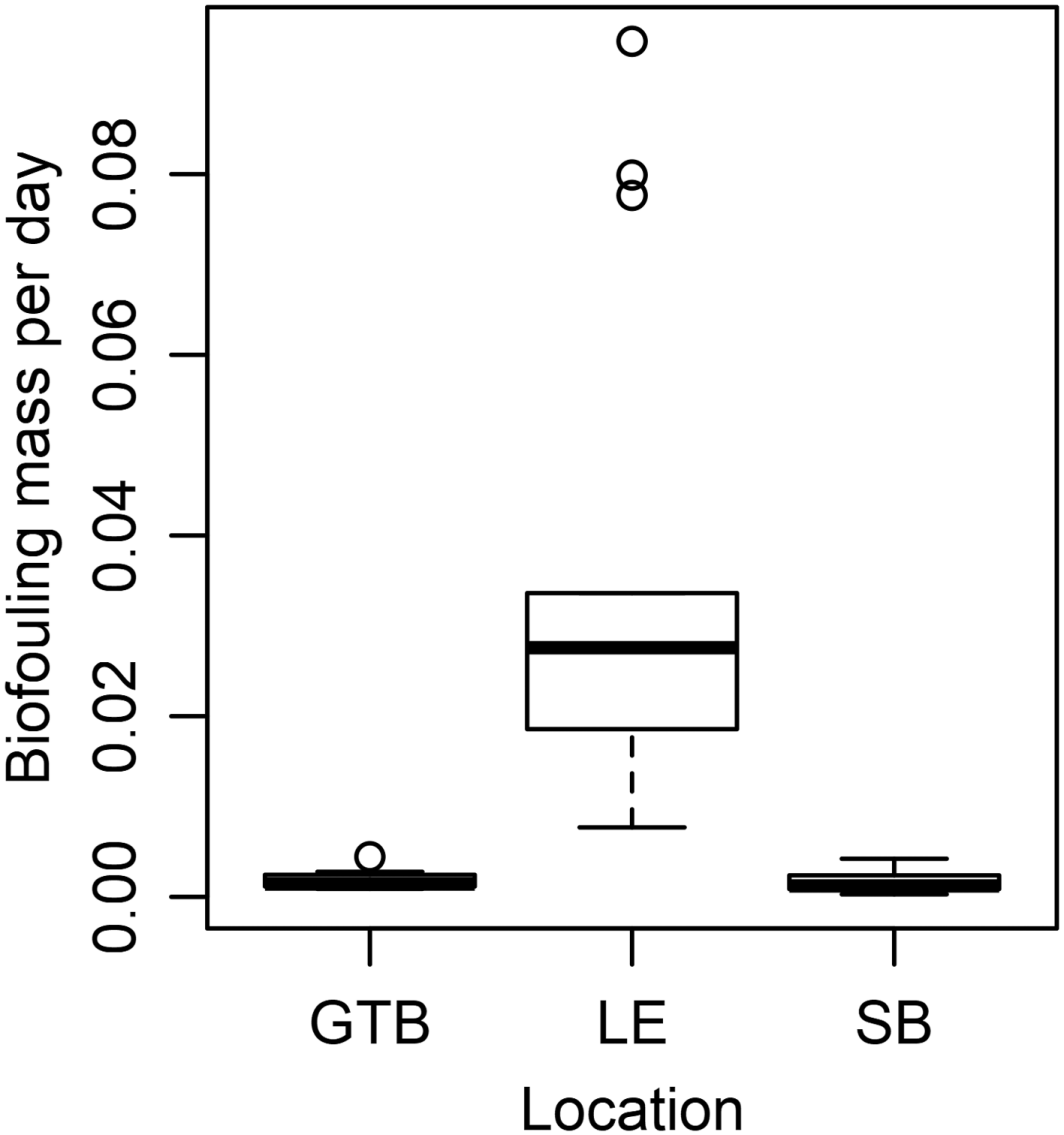
Box‐and‐whisker plot showing biofouling rates (g day^−1^) of individual unionid mussels deployed at open‐water sites in Grand Traverse Bay (GTB; Lake Michigan), western Lake Erie (LE) and Saginaw Bay (SB; Lake Huron) in 2015. GTB—Four sites; LE—Seven sites; SB—Five sites. Each site had 2 individual mussels. The 95% credible interval of the difference between nearshore areas had a magnitude >0.

Sites associated with the Fox and Maumee rivermouths had the highest biofouling rates, but we did not sample analogous habitats in Grand Traverse Bay or Saginaw Bay. The most comparable habitats in Lake Erie to the Grand Traverse Bay or Saginaw Bay sites were the open‐water sites of Lake Erie (using the habitat distinctions identified in Larson, Richardson, Evans, et al., 2016). For this comparison, we used data from 2015, when data were available for all three locations. In habitats more analogous to “open‐water” habitats in Lake Erie, Grand Traverse Bay (median 0.001 g day^−1^) and Saginaw Bay (0.002 g day^−1^) had much lower biofouling rates than Lake Erie (0.027 g day^−1^; Figure 4; Table S3). Dreissenid mussels were not present on unionid shells in Grand Traverse Bay, and only one of the Saginaw Bay sites had dreissenid mussels present on unionid shells (SB3). Small dreissenid mussels were present on equipment at all these sites (mostly on cement blocks, but also a few individuals on buoys and ropes).

**FIGURE 5:**
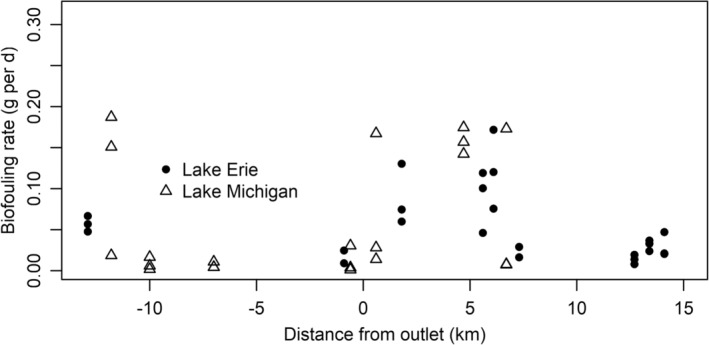
Longitudinal comparison of measured biofouling rates (g day^−1^) along the river‐to‐lake transition in Lake Erie (Maumee rivermouth into Maumee Bay) and Lake Michigan (Fox and Duck Creek rivermouths into Green Bay). Each location had measured biofouling on 2–3 individual mussels.

Green Bay on Lake Michigan had a similar sampling scheme to the one used in the Maumee rivermouth on Lake Erie, where stations occurred in the rivermouth and extended out into the associated shallow water bay (Figure 1). During 2016, in both the Fox‐Green Bay and Maumee‐Maumee Bay transitions, the highest biofouling rate occurred in the area just outside the outlet of the rivermouth (Figure 5, Table S4). A direct comparison between Green Bay and Maumee Bay is limited due to limited sampling within each habitat type. Sites in the channelized portion of the Maumee rivermouth (LE1, LE7, median 0.048 g day^−1^) had higher biofouling than sites in the channelized portion of the Fox and Duck rivermouths (FX1, FX2, FX3, DK1, median 0.009 g day^−1^) and this was a non‐zero difference (Δ Maumee−Fox/Duck = 0.0235 g day^−1^, 95% credible interval = 0.001, 0.081). In terms of the overall longitudinal gradient, the area just outside the outlet (within 10 km of the rivermouth outlet) has the greatest biofouling rates in both systems (Figure 5).

### 3.4 Does biofouling vary with depth and temperature?

**FIGURE 6:**
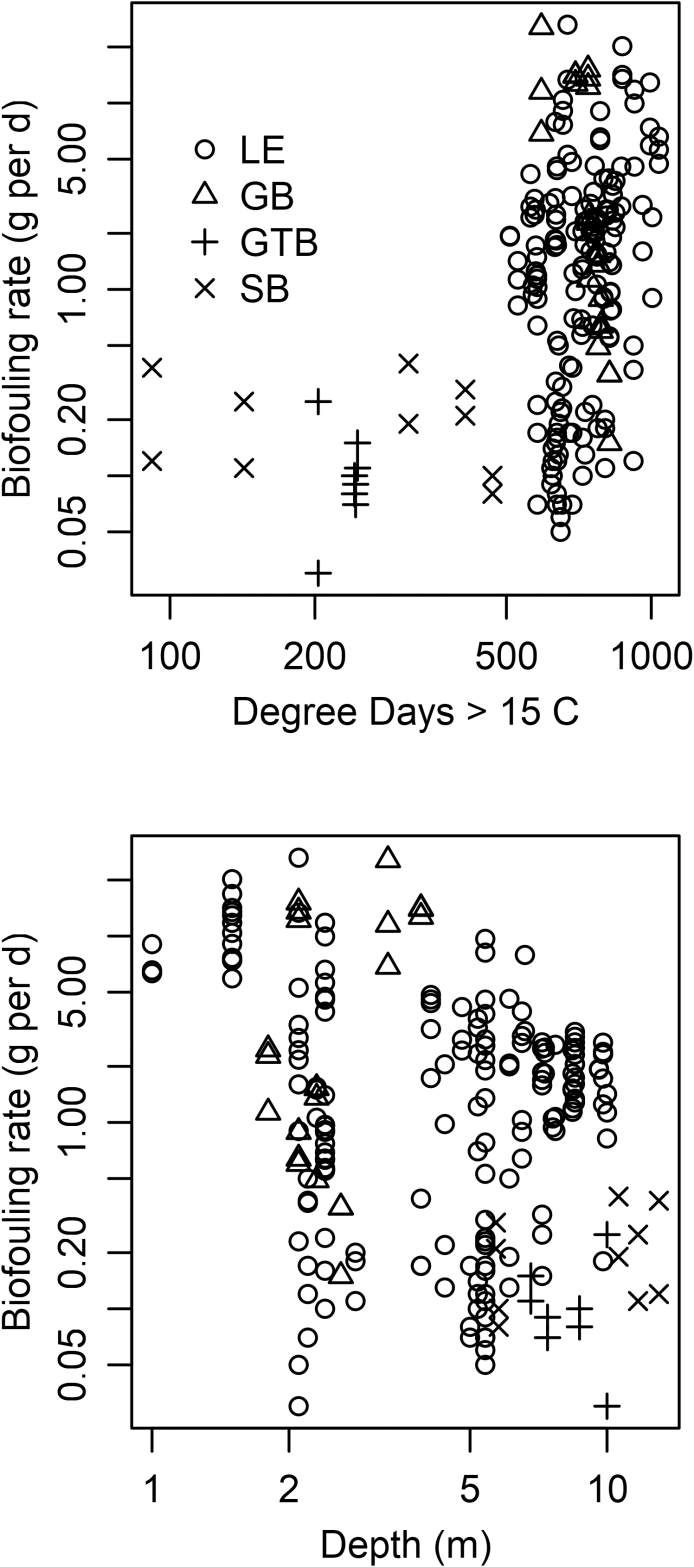
Bivariate plots relating degree days (>15°C) and depth to biofouling rate on unionid mussels suspended in the water column in western Lake Erie/Maumee Bay (LE), Green Bay (Lake Michigan; GB), Grand Traverse Bay (Lake Michigan; GTB), and Saginaw Bay (Lake Huron; SB).

Biofouling was highest in sites in western Lake Erie and Green Bay, and these areas had substantially higher temperatures than our sampling locations in Saginaw Bay and Grand Traverse Bay (Figure 6). Our data indicate that thermal regime (represented by DD15) has a positive statistical association with average biofouling rate within a cage (Spearman's *ρ* = 0.43), but that relationship is clearly non‐linear (Figure 6). The effect of depth is less obvious (Spearman's *ρ* = −0.30; Figure 6). Depth and temperature are relatively strongly correlated (Spearman's *ρ* = −0.62).

## 4 DISCUSSION

### 4.1 Biofouling accumulation is extremely rapid in some locations

Unionid mussels in this study were deployed for only a short time (~3 months) and were free of all biofouling that we could measure at the time of deployment. Yet in that short time, the mass of biofouling organisms had already reached densities that would be expected to kill unionid mussels over time at several sites (i.e., >1.0 ratio of dreissenid mussel mass to unionid mass; Burlakova et al., 2014; Ricciardi et al., 1995; Ricciardi et al., 1996). Although we did not quantify the biomass of different biofouling taxa, previously published data from Hester‐Dendy samplers deployed at the same locations showed the vast majority of variation in dry mass of colonizing organisms was associated with variation in dreissenid mussels (Larson, Richardson, Kennedy, et al., 2016; Larson et al., 2018). In addition, aside from a couple of individual unionid mussels from the Maumee rivermouth that were covered in bryozoan colonies, visually it was obvious that variation in biofouling rate was driven by the abundance of dreissenid mussels (i.e., no mussels were heavily biofouled by anything other than dreissenid mussels). Because the cages were suspended in mid‐water column and no biofouling was present at the start of the experiment, it is likely that almost all of the dreissenid mussels attached to unionid mussels in this study were young‐of‐year juveniles, so this is a better index of veliger settlement density than it is an index of adult dreissenid mussel populations.

A moderate positive correlation was present between biofouling and unionid mussel growth, consistent with earlier positive associations between mussel growth and the biomass of colonizing invertebrates on artificial substrates at these sites (Larson et al., 2018). Although dreissenid mussels are likely to be harmful to unionid mussels at the highest densities, evidence indicates that unionid mussels can persist after zebra mussel invasion (reviewed in Lucy et al., 2013), and this has been the case in Lake Erie as well (Crail et al., 2011). However, virtually all examples of refugia in Lake Erie have been associated with some environmental characteristic that is hypothesized to reduce dreissenid mussel biofouling (reviewed in Bossenbroek et al., 2018). Still, Bossenbroek et al. (2018) found low and moderate densities of dreissenid mussels were associated with higher likelihood of unionid mussel presence (compared to high densities or the absence of dreissenid mussels). We hypothesize that the moderate positive correlation between biofouling and unionid mussel growth we observed is probably related to common factors influencing the growth of both unionid mussels and dreissenid mussels. For example, both dreissenid mussel and unionid mussels may grow better when higher‐quality food resources are available (Larson, Richardson, Evans, et al., 2016) and may grow better in warmer temperatures.

### 4.2 The possibility of an open lake refugia

Observations of unionid mussels in western Lake Erie and Lake St. Clair in “refugia” have demonstrated co‐occurrence with dreissenid mussels occurs at some spatial scales (Bryan et al., 2013; Crail et al., 2011; Lucy et al., 2013). However, all of these refugia are associated with mechanisms that reduce dreissenid mussel survival or attachment (discussed in Bossenbroek et al., 2018). For example, “veliger shadows,” nearshore dewatering and ice scour, soft sediments, and wave action are all hypothesized to promote unionid mussel co‐occurrence with dreissenid mussels via reduced biofouling. Based on modeling, less than 1% of the nearshore of Lake Erie and Lake Ontario may be suitable for unionid mussels (65.5 km^2^ of Lake Erie; Bossenbroek et al., 2018). This makes the overall unionid mussel community extremely vulnerable to further habitat degradation and localized pollution events.

Our data on biofouling indicate that these limited nearshore areas are not the only places where dreissenid mussel biofouling is low. Larson, Richardson, Kennedy, et al. (2016) used 0.010 g per mussel per day as a threshold for harmful levels of biofouling on unionid mussels, as that was the highest biofouling rate observed without dreissenid mussels. In 2014, hundreds of km^2^ of the western basin of Lake Erie had a biofouling rate below that, and 315 km^2^ had both a very low biofouling rate *and* soft substrates, ~5× the nearshore area that has been identified as suitable habitat (Larson, Richardson, Kennedy, et al., 2016). Based on the data that we have added in this analysis, the total area below that threshold would be less in 2015 and 2016 (as 2014 had lower biofouling than the other years). However, this threshold is not based on experimental work to estimate lethal biofouling, and the actual threshold at which lethal biofouling occurs could be much different.

Several species that are known to occur in Lake Erie would seem to be ideal candidates for occupying or re‐colonization of these open‐water habitats. For example, *Leptodea fragilis* and *Pyganodon grandis* are often found in Lake Erie nearshore habitats and are considered generalist species with a wide range of fish hosts (Crail et al., 2011; Haag, 2012). Our own experiments with caged unionid mussels (*L. siliquoidea*) demonstrate that unionid mussels can survive and grow in these locations, even in highly artificial conditions and after handling (although at a slower rate than in warmer, nearshore sites; Larson, Richardson, Kennedy, et al., 2016). We are not aware of any systematic investigation of these open‐water habitats for unionid mussels (i.e., between 3 and 10 m deep). For many years, soft substrates were surveyed for mayfly nymphs (sampling scheme described in Stapanian et al., 2017), and during the surveys, unionid mussels were occasionally observed in sediment samples, but those observations were not documented quantitatively (Donald Schloesser, U.S. Geological Survey, written comm. 2016). Even if unionid mussels are not present now, that could be due to heavy mortality during the early part of the dreissenid mussel invasion, and these areas could be primed for natural or artificial recolonization in the future (Burlakova et al., 2014; Karatayev et al., 2014; Patterson et al., 2018).

### 4.3 Nearshore areas near productive rivermouths are favorable for high biofouling rates

Compared to Lake Erie, Saginaw Bay and Grand Traverse Bay had very low biofouling rates. Dreissenid mussels have been implicated in the restructuring of the plankton community within Saginaw Bay and Lake Michigan (Bunnell et al., 2014; Evans et al., 2011; Vanderploeg et al., 2001, 2010), but even though dreissenids were clearly present in these bays, they were not observed in high numbers on our equipment and only a few were observed in Saginaw Bay on our caged unionid mussels. Accumulation of dreissenid mussels on these live unionids was therefore nearly zero, although presumably some low rate of accumulation would occur over longer time periods. The sites we sampled in Grand Traverse Bay and Saginaw Bay appear to have very different environmental conditions than Lake Erie habitats. This was obvious with temperature and the depth of most sites, but was also apparent visually. Turbidity in Lake Erie was almost always very high, and we could not see buoys or cages sitting on the bottom even when the depth was only 2–3 m. By contrast, we could clearly see cages and buoys on the bottom in both Grand Traverse Bay and Saginaw Bay at depths of ~10 m. These clearly observable differences demonstrate that many environmental factors are likely to differ between these nearshore areas, and therefore isolating a causative factor is difficult. We did observe a moderate correlation between biofouling and temperature across all sites, driven mostly by variation between nearshore areas (i.e., Lake Erie and Grand Traverse Bay). Other factors, such as water movement transporting veligers, may be a more important driver of variation in biofouling within a particular nearshore area (McGoldrick et al., 2009; Trebitz et al., 2019). The areas with the highest biofouling were those where channelized rivermouth areas intersected with lentic waters, a zone often associated with the deposition of sediment and high primary productivity (Larson et al., 2013; Larson, Frost, Vallazza, et al., 2016).

In terms of temperature, depth, and turbidity, the Fox rivermouth/Green Bay area of Lake Michigan was similar to the Maumee rivermouth/Maumee Bay area of Lake Erie, and biofouling was also high in the Fox rivermouth/Green Bay area. Biofouling was highest in the area immediately outside the channelized portion of the Fox and Maumee rivermouths. This is the same general area where unionid growth is greatest and where the highest quality food seems to be available in Lake Erie (Larson, Richardson, Evans, et al., 2016). Both Green Bay and Maumee Bay are fed by large rivers draining agricultural watersheds, and these embayments are dominated by those inputs. Furthermore, rivermouths with strong agricultural influences appear more likely to have big increases in chlorophyll *a* before reaching the lake, meaning these rivermouths are exporting high‐quality food resources into the nearshore zone (Larson, Frost, Vallazza, et al., 2016). Although these characteristics also describe conditions near the Saginaw rivermouth, our sampling in Saginaw Bay was much farther from the mouth of the Saginaw River. The area of influence for the Maumee rivermouth for biofouling appears to extend only about 10–15 km, after which biofouling rates are much lower, and other portions of Lake Erie appear to lack dreissenid mussel biofouling past a certain depth (Karatayev et al., 2018). This is also the area where flowing lotic waters are slowed by mixing with lentic waters, a zone often associated with the deposition of sediment and high primary productivity (Larson, Frost, Vallazza, et al., 2016; Lucy et al., 2013).

## 5 CONCLUSIONS

In terms of unionid mussel conservation and restoration, a potentially important issue is whether open‐water areas with low biofouling are suitable for unionid mussel conservation. In a study of nearshore areas, Bossenbroek et al. (2018) found that areas with low and moderate dreissenid mussel densities were more likely to have unionid mussels present than areas without dreissenid mussels. Although there is no mechanistic reason to think that dreissenid mussels promote unionid mussels, both taxa feed on similar resources and are likely impaired by similar stressors (Baker & Levinton, 2003; Larson et al., 2018). Therefore, open‐water areas with low biofouling rates may also be poorer habitat for unionid mussels. However, given the areal extent of these areas with low biofouling rates, it may be worthwhile for future researchers and mussel conservation experts to assess open‐water habitats for their potential to function as habitat for unionid mussels.

## AUTHOR CONTRIBUTIONS

**James H. Larson:** Conceptualization (lead); data curation (equal); formal analysis (lead); funding acquisition (lead); investigation (lead); methodology (equal); project administration (lead); supervision (lead); visualization (lead); writing – original draft (lead); writing – review and editing (equal). **Sean W. Bailey:** Investigation (equal); methodology (equal); writing – review and editing (equal). **Mary Anne Evans:** Conceptualization (equal); formal analysis (equal); methodology (equal); resources (equal); writing – review and editing (equal).

## FUNDING INFORMATION

This research was funded by the U.S. Geological Survey (Ecosystem Mission Area) and the Great Lakes Restoration Initiative.

## CONFLICT OF INTEREST

The authors declare that they have no conflict of interest.

## Supporting information


Figure S1

Appendix S1

## Data Availability

All data associated with this paper, along with example R code that describes the statistical analysis and data processing steps used in this manuscript, are available from doi: 10.5066/P9RL5BU4 (Larson et al., 2022).
