# Supplementary figures and images for: Biofouling of a unionid mussel by dreissenid mussels in nearshore zones of the Great Lakes
Source: Ecol Evol. 2022 Dec 13;12(12):e9557. doi: 10.1002/ece3.9557 (PMC9745470; doi:10.1002/ece3.9557)

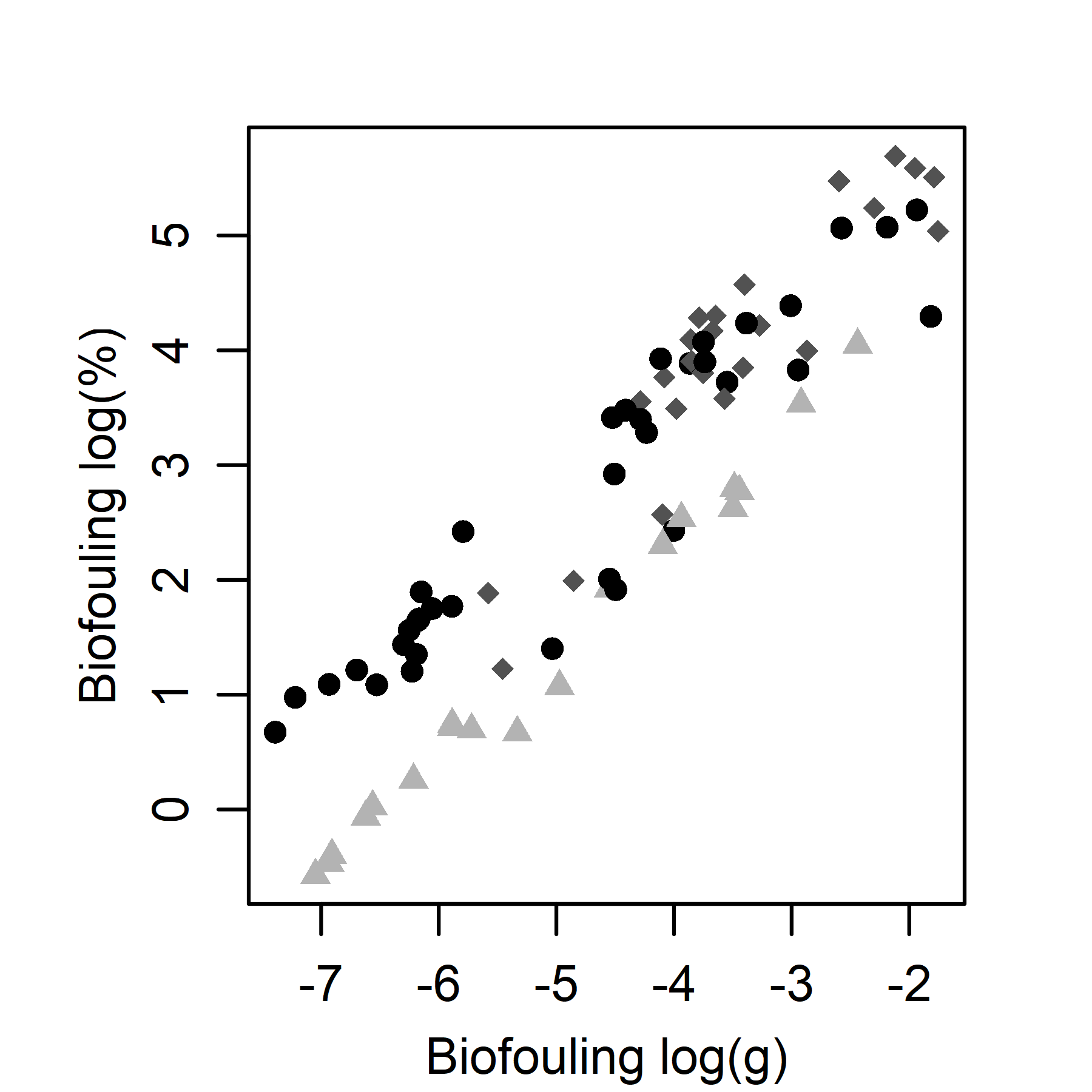

Supplement: Supplementary file 1 — Figure S1 [file ECE3-12-e9557-s001.tif]
