# Supplementary material for: Biofouling of a unionid mussel by dreissenid mussels in nearshore zones of the Great Lakes
Source: Ecol Evol. 2022 Dec 13;12(12):e9557. doi: 10.1002/ece3.9557 (PMC9745470; doi:10.1002/ece3.9557)

Table S1. Median, mean, standard deviation, 25 and 75 percent quantiles of biofouling rate (g d^-1^) in cages deployed in the western basin of Lake Erie (LE), Grand Traverse Bay (GTB; Lake Michigan), Saginaw Bay (SB; Lake Huron), Green Bay (GB; Lake Michigan), and two tributaries to Green Bay, the Fox River (FX) and Duck Creek (DK).

| **Site - Year** | **Median Biofouling Rate** | **Mean (st dev)**  **Biofouling Rate** | **25-75 quartiles of**  **Biofouling Rate** | **Ratio of biofouling mass to mussel mass** |
| --- | --- | --- | --- | --- |
| LE1 - 2014 | 0.1627 | 0.1827 (0.1295) | 0.1135, 0.2419 | 0.90 (0.72) |
| LE1 - 2015 | 0.0303 | 0.0303 (0.0091) | 0.0271, 0.0335 | 0.14 (0.05) |
| LE1 - 2016 | 0.0169 | 0.0169 (0.0110) | 0.0130, 0.0208 | 0.43 (0.08) |
| LE2 - 2014 | 0.0183 | 0.0167 (0.0033) | 0.0156, 0.0186 | 0.15 (0.08) |
| LE6 - 2014 | 0.0065 | 0.0097 (0.0057) | 0.0065, 0.0114 | 0.05 (0.02) |
| LE7 - 2014 | 0.0112 | 0.0105 (0.0013) | 0.0101, 0.0112 | 0.06 (0.01) |
| LE7 - 2015 | 0.0048 | 0.0048 (0.0031) | 0.0037, 0.0059 | 0.02 (0.01) |
| LE7 - 2016 | 0.0568 | 0.0570 (0.0095) | 0.0522, 0.0617 | 0.50 (0.18) |
| LE8 - 2014 | 0.0106 | 0.0222 (0.0203) | 0.0105, 0.0281 | 0.15 (0.13) |
| LE12 - 2014 | 0.0762 | 0.0850 (0.0176) | 0.0748, 0.0907 | 1.66 (0.42) |
| LE13 - 2014 | 0.1121 | 0.1116 (0.0170) | 0.1033, 0.1202 | 1.59 (0.22) |
| LE13 - 2016 | 0.1202 | 0.1225 (0.0481) | 0.0979, 0.1460 | 2.53 (0.83) |
| LE14 - 2014 | 0.0006 | 0.0013 (0.0014) | 0.0005, 0.0017 | 0.05 (0.05) |
| LE14 - 2016 | 0.0227 | 0.0227 (0.0089) | 0.0195, 0.0258 | 0.73 (0.15) |
| LE17 - 2014 | 0.0018 | 0.0017 (0.0005) | 0.0015, 0.0019 | 0.04 (0.01) |
| LE18 - 2014 | 0.1444 | 0.1627 (0.0378) | 0.1409, 0.1753 | 1.83 (0.24) |
| LE18 - 2016 | 0.0745 | 0.0882 (0.0371) | 0.0672, 0.1024 | 2.22 (0.64) |
| LE20 - 2014 | 0.0020 | 0.0037 (0.0036) | 0.0016, 0.0049 | 0.07 (0.07) |
| LE20 - 2015 | 0.0069 | 0.0069 (0.0009) | 0.0066, 0.0073 | 0.03 (<0.01) |
| LE20 - 2016 | 0.1005 | 0.0886 (0.0380) | 0.0733, 0.1098 | 1.92 (0.77) |
| LE21 - 2014 | 0.0012 | 0.0012 (0.0002) | 0.0012, 0.0013 | 0.03 (<0.01) |
| LE21 - 2016 | 0.0138 | 0.0137 (0.0057) | 0.0109, 0.0166 | 0.37 (0.08) |
| LE22 - 2014 | 0.0023 | 0.0034 (0.0025) | 0.0020, 0.0043 | 0.07 (0.02) |
| LE22 - 2016 | 0.0211 | 0.0295 (0.0152) | 0.0208, 0.0341 | 0.69 (0.21) |
| LE23 - 2014 | 0.0015 | 0.0015 (0.0002) | 0.0013, 0.0016 | 0.03 (<0.01) |
| LE23 - 2015 | 0.0105 | 0.0105 (0.0040) | 0.0091, 0.0120 | 0.07 (0.05) |
| LE23 - 2016 | 0.0330 | 0.0312 (0.0066) | 0.0284, 0.0348 | 0.48 (0.10) |
| LE24 - 2014 | 0.0007 | 0.0013 (0.0012) | 0.0007, 0.0017 | 0.05 (0.05) |
| LE24 - 2016 | 0.0261 | 0.0288 (0.0087) | 0.0239, 0.0323 | 0.69 (0.17) |
| LE26 - 2014 | 0.0210 | 0.0230 (0.0089) | 0.0181, 0.0268 | 0.53 (0.11) |
| LE26 - 2016 | 0.0234 | 0.0165 (0.0127) | 0.0126, 0.0238 | 0.37 (0.32) |
| LE28 - 2014 | 0.0021 | 0.0021 (0.0008) | 0.0019, 0.0024 | 0.07 (0.02) |
| LE28 - 2015 | 0.0166 | 0.0166 (0.0083) | 0.0137, 0.0196 | 0.10 (0.05) |
| LE29 - 2014 | 0.0030 | 0.0029 (0.0010) | 0.0024, 0.0035 | 0.09 (0.04) |
| LE29 - 2015 | 0.0195 | 0.0195 (0.0013) | 0.0190, 0.0200 | 0.13 (0.01) |
| LE29 - 2016 | 0.0254 | 0.0230 (0.0060) | 0.0208, 0.0264 | 0.56 (0.17) |
| LE30 - 2014 | 0.0145 | 0.0155 (0.0023) | 0.0142, 0.0163 | 0.30 (0.11) |
| LE30 - 2016 | 0.0187 | 0.0197 (0.0048) | 0.0171, 0.0218 | 0.33 (<0.01) |
| LE31 - 2014 | 0.0235 | 0.0235 (0.0002) | 0.0235, 0.0236 | 0.59 (0.04) |
| LE32 - 2014 | 0.0138 | 0.0137 (0.0037) | 0.0119, 0.0155 | 0.26 (0.07) |
| LE33 - 2014 | 0.0021 | 0.0020 (0.0010) | 0.0015, 0.0025 | 0.05 (0.02) |
| LE33 - 2015 | 0.0873 | 0.0873 (0.0105) | 0.0836, 0.0910 | 0.57 (0.11) |
| LE33 - 2016 | 0.0379 | 0.0379 (0.0129) | 0.0333, 0.0424 | 0.68 (0.02) |
| LE34 - 2015 | 0.0540 | 0.0540 (NA) | 0.0421, 0.0658 | 0.34 (0.14) |
| LE35 - 2014 | 0.0163 | 0.0190 (0.0052) | 0.0160, 0.0207 | 0.45 (0.13) |
| LE35 - 2015 | 0.0306 | 0.0306 (0.0043) | 0.0291, 0.0321 | 0.16 (0.05) |
| LE35 - 2016 | 0.0212 | 0.0225 (0.0066) | 0.0189, 0.0255 | 0.49 (0.02) |
| LE36 - 2014 | 0.0109 | 0.0104 (0.0024) | 0.0093, 0.0117 | 0.25 (0.09) |
| LE36 - 2015 | 0.032 | Only 1 mussel |  | 0.16 |
| LE36 - 2016 | 0.0334 | 0.0334 (0.0091) | 0.0302, 0.0366 | 0.97 (0.04) |
| LE50 - 2014 | 0.0019 | 0.0033 (0.0027) | 0.0017, 0.0042 | 0.10 (0.08) |
| LE51 - 2014 | 0.002 | 0.0029 (0.0015) | 0.002, 0.0034 | 0.06 (0.04) |
| LE52 - 2014 | 0.011 | 0.0157 (0.0097) | 0.0102, 0.019 | 0.22 (0.09) |
| LE53 - 2014 | 0.0028 | 0.0024 (0.0014) | 0.0018, 0.0032 | 0.05 (0.02) |
| LE54 - 2014 | 0.001 | 0.0013 (0.0007) | 0.0009, 0.0015 | 0.04 (0.02) |
| LE55 - 2014 | 0.0238 | 0.0232 (0.0049) | 0.0209, 0.0258 | 0.46 (0.09) |
| LE56 - 2014 | 0.0288 | 0.0273 (0.0044) | 0.0255, 0.0298 | 0.41 (0.04) |
| LE60 - 2014 | 0.0121 | 0.0121 (0.0009) | 0.0118, 0.0125 | 0.32 (0.05) |
| LE61 - 2014 | 0.0527 | 0.0429 (0.0191) | 0.0367, 0.0539 | 0.51 (0.27) |
| LE62 - 2014 | 0.0494 | 0.0494 (0.0145) | 0.0443, 0.0545 | 0.81 (0.06) |
| LE63 - 2014 | 0.0021 | 0.0026 (0.002) | 0.0015, 0.0034 | 0.07 (0.06) |
| LE63 - 2016 | 0.0038 | 0.0034 (0.002) | 0.0025, 0.0044 | 0.08 (0.05) |
| LE64 - 2014 | 0.0339 | 0.0381 (0.0112) | 0.0318, 0.0423 | 0.70 (0.08) |
| DK1 - 2016 | 0.0281 | 0.0242 (0.009) | 0.021, 0.0293 | 0.74 (0.90) |
| FX0 - 2016 | 0.1673 | 0.1684 (0.0182) | 0.1591, 0.1772 | 2.53 (0.13) |
| FX1 - 2016 | 0.0166 | 0.0137 (0.0068) | 0.0113, 0.0176 | 0.11 (0.07) |
| FX2 - 2016 | 0.0043 | 0.0035 (0.0014) | 0.003, 0.0043 | 0.03 (0.01) |
| FX3 - 2016 | 0.0078 | 0.0087 (0.0019) | 0.0076, 0.0093 | 0.08 (0.04) |
| GB25 - 2016 | 0.173 | 0.1681 (0.0098) | 0.1649, 0.1738 | 1.53 (0.03) |
| GB32 - 2016 | 0.1422 | 0.1809 (0.1203) | 0.1135, 0.229 | 2.72 (0.27) |
| GTB1 - 2015 | 0.0009 | 0.0009 (0.0002) | 0.0008, 0.0009 | 0.01 (<0.01) |
| GTB2 - 2015 | 0.0013 | 0.0013 (0.0015) | 0.0008, 0.0019 | 0.01 (0.01) |
| GTB3 - 2015 | 0.0014 | 0.0014 (0.0003) | 0.0013, 0.0015 | 0.01 (<0.01) |
| GTB4 - 2015 | 0.001 | 0.001 (0.0002) | 0.0009, 0.001 | 0.01 (<0.01) |
| SB1 - 2015 | 0.002 | 0.002 (0.0011) | 0.0016, 0.0024 | 0.01 (<0.01) |
| SB2 - 2015 | 0.0028 | 0.0028 (0.002) | 0.0021, 0.0035 | 0.02 (0.02) |
| SB3 - 2015 | 0.0028 | 0.0028 (0.0006) | 0.0026, 0.003 | 0.02 (<0.01) |
| SB4 - 2015 | 0.0033 | 0.0033 (0.0016) | 0.0027, 0.0039 | 0.02 (<0.01) |
| SB5 - 2015 | 0.001 | 0.001 (0.0002) | 0.0009, 0.0011 | 0.01 (<0.01) |

Table S2. Average *Lampsilis siliquoidea* biofouling rate in wet mass (g d^-1^) over the growing season at sites sampled over three years (Sites LE1, LE7, LE20, LE23, LE29, LE33, LE35, and LE36). Difference between individual years is also reported (with 95% credible interval), with negative indicating an increase from the earlier year to the later year. Averages are the mean of the log-normal distribution estimated from all individuals each year (with 95% credible intervals).

| Year | Mean biofouling rate for log-normal distribution (95% credible interval) |
| --- | --- |
| 2014 | 0.007 (0.004, 0.013) |
| 2015 | 0.018 (0.010, 0.031) |
| 2016 | 0.034 (0.025, 0.044) |
| Δ2014 – 2015 | -0.011 (-0.025, -0.001) |
| Δ2015 – 2016 | -0.015 (-0.029, -0.0003) |
| Δ2014 – 2016 | -0.026 (-0.038, -0.016) |

Table S3. Comparison of average *Lampsilis siliquoidea* biofouling rate in wet mass (g d^-1^) over the 2015 growing season at open water sites in Lake Erie (Sites LE23, LE28, LE29, LE33, LE35, and LE36). Difference between individual bays is also reported (with 95% credible interval), with negative indicating an increase from the first to second bay listed. Averages are the mean of the log-normal distribution estimated from all individuals each year (with 95% credible intervals).

| Year | Mean biofouling rate for log-normal distribution (95% credible interval) |
| --- | --- |
| Western Lake Erie | 0.027 (0.017, 0.043) |
| Grand Traverse Bay (Lake Michigan) | 0.001 (0.0006, 0.0017) |
| Saginaw Bay (Lake Huron) | 0.002 (0.001, 0.003) |
| ΔLake Erie – Grand Traverse Bay | 0.026 (0.016, 0.042) |
| ΔLake Erie – Saginaw Bay | 0.025 (0.015, 0.041) |
| ΔGrand Traverse Bay – Saginaw Bay | -0.0011 (-0.0022, -0.0001) |

Table S4. Longitudinal comparison of biofouling in the Maumee rivermouth and Maumee Bay (Lake Erie, LE) compared to biofouling in the Fox rivermouth, Duck Creek rivermouth, and Green Bay (FX, DK and GB, respectively, in Lake Michigan) during 2016. Biofouling is reported as the median and standard deviation (g d^-1^) of 2-3 individual mussels at each site. Distance from the outlet refers to the approximate distance (in km) from the site to the location where the rivermouth intersects with the coast (near 41.697791° N, 83.463676° W for the Maumee River; 44.539875°N, 88.004202°W for the Fox River, 44.564368°N, 88.041253°W for Duck Creek). Negative values indicate upstream distance and positive values indicate distances into the associated bay. Site DK1 is in the rivermouth of Duck Creek, which is another tributary to Green Bay and is included here for comparison to FX0 and LE1.

| Lake Erie | Distance | Biofouling | Lake Michigan | Distance | Biofouling |
| --- | --- | --- | --- | --- | --- |
| LE7 | -12.9 | 0.057 (0.009) |  |  |  |
|  |  |  | FX1 | -11.8 | 0.017 (0.007) |
|  |  |  | FX2 | -10.0 | 0.004 (0.001) |
|  |  |  | FX3 | -7.0 | 0.008 (0.002) |
| LE1 | -0.9 | 0.017 (0.011) |  |  |  |
|  |  |  | DK1 | -0.6 | 0.028 (0.009) |
|  |  |  | FX0 | 0.6 | 0.167 (0.018) |
| LE18 | 1.8 | 0.075 (0.037) |  |  |  |
|  |  |  | GB32 | 4.7 | 0.142 (0.120) |
| LE20 | 5.6 | 0.100 (0.038) |  |  |  |
| LE13 | 6.1 | 0.120 (0.048) |  |  |  |
|  |  |  | GB25 | 6.7 | 0.173 (0.010) |
| LE14 | 7.3 | 0.023 (0.009) |  |  |  |
| LE21 | 12.7 | 0.014 (0.006) |  |  |  |
| LE23 | 13.4 | 0.033 (0.007) |  |  |  |
| LE22 | 14.1 | 0.021 (0.015) |  |  |  |

Figure S1. Comparison of biofouling rate calculated as a percentage of the unionid mass (%) to biofouling as g per day of exposure (g). Both methods of expressing biofouling are log-transformed.


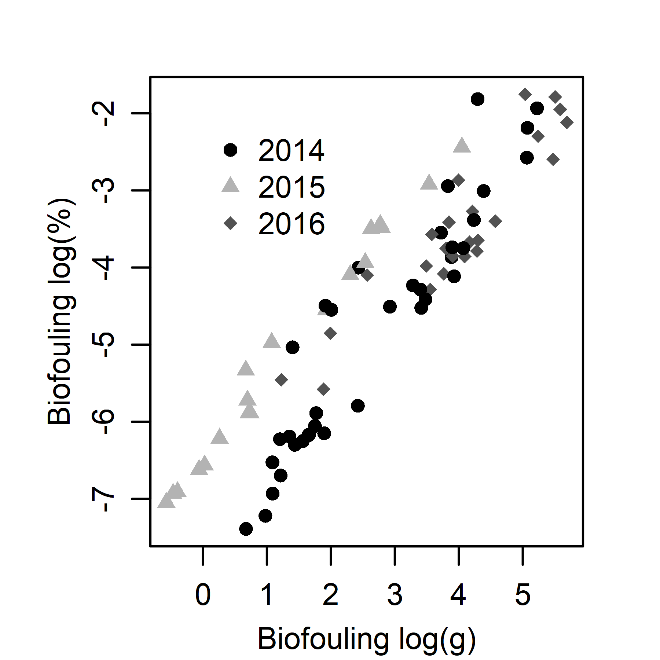

Supplement: Supplementary file 2 — Appendix S1 [file ECE3-12-e9557-s002.docx]
